# Bacterial secretion systems: Networks of pathogenic regulation and adaptation in mycobacteria and beyond

**DOI:** 10.1371/journal.ppat.1010610

**Published:** 2022-07-14

**Authors:** Kathleen R. Nicholson, Patricia A. Champion

**Affiliations:** Eck Institute for Global Health, Department of Biological Sciences, University of Notre Dame, Notre Dame, Indiana, United States of America; University of Geneva Faculty of Medicine: Universite de Geneve Faculte de Medecine, SWITZERLAND

## Introduction

Bacteria transport proteins to diverse intracellular or extracellular locations to ensure proper function. Proteins are localized intracellularly to the cytoplasm or the periplasm or embedded in membranes. Extracellular proteins are localized to the bacterial surface, secreted into the environment, or directly transported into bacterial or host cells. Protein transport systems can be general or specialized. Recently, there have been several advances in our understanding of specialized protein secretion in pathogenic mycobacteria. Here, we will review broad themes across bacterial secretion systems, highlighting that, although gram-negative and Mycobacterial/Gram-positive specialized secretion systems are unrelated, they share conserved themes of regulation and environmental sensing.

### 1. Protein transport is essential for bacterial physiology

General secretion systems, including the Sec and twin arginine translocation (Tat) systems, are universal in bacteria. Sec and Tat systems export proteins across the cytoplasmic membrane [1]. Proteins exported into the periplasm by Sec or Tat can be secreted extracellularly by specialized secretion systems in a 2-step mechanism. Some specialized systems secrete protein substrates in a single step from the cytoplasm out of the cell, independently of Sec and Tat [2].

Gram-negative bacteria have 2 membranes (diderms). Proteins and solutes transit across both membranes [3]. Gram-negative specialized secretion systems include Type 1 to 6 and Type 8 to 10 systems that transport proteins across membranes [4–6]. Porins promote solute transport across the outer membrane (OM). Gram-negative bacteria have unique assembly machinery (β-barrel assembly machinery, BAM) that reside within and localize porins to the OM [7].

Mycobacteria are also diderms, but have a unique OM compared to gram-negative bacteria [8–10]. The mycobacterial OM (MOM) requires distinct mechanisms to maintain impermeability while allowing protein, metal, and solute transport. Mycobacteria do not have obvious BAM complexes, and the pathogenic species lack porins. Mycobacteria have Type VII specialized secretion systems (T7SS) [11,12]. Mycobacterial T7SSs (ESX or ESAT-6-systems) are genetically unrelated to specialized gram-negative systems. Interestingly, although gram-positive bacteria are monoderms, T7SS are widespread and are the topic of study in several species [13].

The T7SS that transits the cytoplasmic membrane has been well described [14–17]. The T7SS protein transporter spanning the MOM has not been identified. Similar to T3SS, T4SS and T6SS, several studies suggest that there are extracytoplasmic components of T7SS that may include known proteins transported by these systems. Recent work illustrated that PE/PPE proteins, which are known T7 substrates localized to the MOM, likely function in transport [18–20]. Several studies suggest PE/PPE proteins could function similar to BAM complexes for protein localization or like porins for solute transport, providing a bidirectional route of transport across the MOM [19–21]. Another family of T7-dependent secreted proteins, called WXG proteins [22], are also widely associated with ESX systems and may likewise play a role in protein transport. Accordingly, WXG proteins associated with T7SS are often required for the secretion of other substrates [11,23–26].

### 2. Specialized secretion systems drive pathogenesis in diverse ways

Secretion systems are a critical interface between host and bacterial pathogen. Some secretion systems promote adaptation to the host environment, while others promote immune evasion. Despite the functional diversity of secretion systems, many are structurally homologous [27]. Some pathogens encode for a single specialized secretion system that serves multiple roles required for pathogenesis. Type IV systems are classified into 2 distinct subtypes based on the type of substrate they secrete (DNA or protein). *Legionella* and *Coxiella* pathogenesis requires a single genomically encoded T4BSS which, through the secretion of numerous protein substrates, supports bacterial replication and causes host cell death [28–32]. This phenomenon appears to be specific to intracellular pathogens with the T4BSS, as T4ASS promote nonpathogenic processes including conjugation [33]. However, in the absence of a functional T4BSS, the T4ASS in *Legionella* is sufficient for pathogenesis under conditions mimicking aquatic environments [34]. Other bacteria have multiple copies of a single type of secretion system encoded in the genome or on plasmids. Each secretion system serves a specific role during infection. For example, *Salmonella* has 2 T3SS that mediate bacterial invasion and intracellular survival, respectively [35–38]. Pathogenic *Mycobacterium* have up to 5 genome encoded T7SS, each with discrete roles in phagosomal escape, phagosomal repair, and macrophage toxification [23,24,39–43].

### 3. Some specialized secretion systems are essential

While many specialized secretion systems are required for survival in the host, some specialized secretion systems are also essential for general bacterial physiology. Several mycobacterial T7SS are also essential for mycobacterial survival during in vitro growth because they are required for iron uptake and maintaining envelope impermeability [19,42–46].

An opposing example of this phenomenon is the accessory Sec system, SecA2, of mycobacteria and some gram-positive bacteria [47]. The Sec system is essential for cell viability because it mediates extracytoplasmic protein localization [1]. The accessory system SecA2 is dispensable for bacterial viability in vitro but is essential for virulence [48]. Across the bacterial kingdom, secretion systems that are specialized or general, essential, and nonessential fill pathogenic and physiological niches necessary for survival.

### 4. Secretion is a controlled and regulated process

Although secretion systems differ in structure and function, they employ common regulatory themes at the molecular level. Protein transporters are made up of secretion system components that are transcriptionally regulated in gram-negative bacteria [49,50]. Likewise, networks of transcriptional regulation modulate the amount of substrate production. Transcriptional control of substrate levels for T3SS in several pathogens depends upon feedback regulation that responds to several intrinsic secretory cues including substrate concentration, localization, and protein transporter assembly or function [51–58]. Feedback regulation was recently discovered in the T7SS (ESX-1) of pathogenic mycobacteria. At least 2 ESX-1–associated transcription factors control substrate gene expression in response to the presence or absence of a protein transporter in the cytoplasmic membrane [54–58]. These regulatory systems likely assure that appropriate levels of substrates are maintained during active secretion and prevent production and accumulation of protein substrates when there is no functional pathway for export [59].

In addition to feedback regulation, protein secretion systems, for example, T3SS and T7SS, are often controlled by extrinsic regulators including two-component systems and other transcription factors [53,60,61].

Secretion systems are also controlled posttranslationally. For example, in Type III secretion injectisome systems (T3SS) of gram-negative pathogens, sequential transport of proteins—or “secretion hierarchy”—is well documented [62]. In hierarchical secretion, secreted components of the protein transporter and substrates are transported sequentially. Substrates are localized to the protein transporter via varying affinities to the transporter or secretion chaperones, which determines secretion hierarchy [62]. Disruptions in secretion hierarchy via loss of individual proteins from machinery or substrates results in a loss of translocon pore formation and a functional secretory apparatus. Work in mycobacteria has shown that loss of individual ESX-1 substrates results in a continuum of secretion defects [25], suggesting substrates may be secreted hierarchically.

### 5. Secretion systems sense and respond to their environments

Bacteria encounter and adapt to a variety of extracellular environments. Bacteria use secretion systems to sense and respond to specific extracellular stimuli that relay information to the bacteria about their environmental context. For example, Type VI secretion systems (T6SSs) in *Vibrio cholerae* sense and respond to the host environment, including bile acids [63]. Environmental sensing by T7SS of pathogenic mycobacteria has not yet been observed, but responses to environmental cues may explain the discrete spatiotemporal functions of ESX secretion systems during infection. Considering that T7SSs function in a variety of niches within the macrophage, it is likely that they are attuned to the distinct microenvironments of the phagosome and the cytoplasm. Indeed, the ESX-1 system functions in the phagosome to promote cytoplasmic access for pathogenic mycobacteria [64]. Since the ESX-1 system controls gene expression, this T7SS may indeed serve to sense and respond to cytoplasmic exposure [54,55,57].

## Conclusions

**Fig 1:**
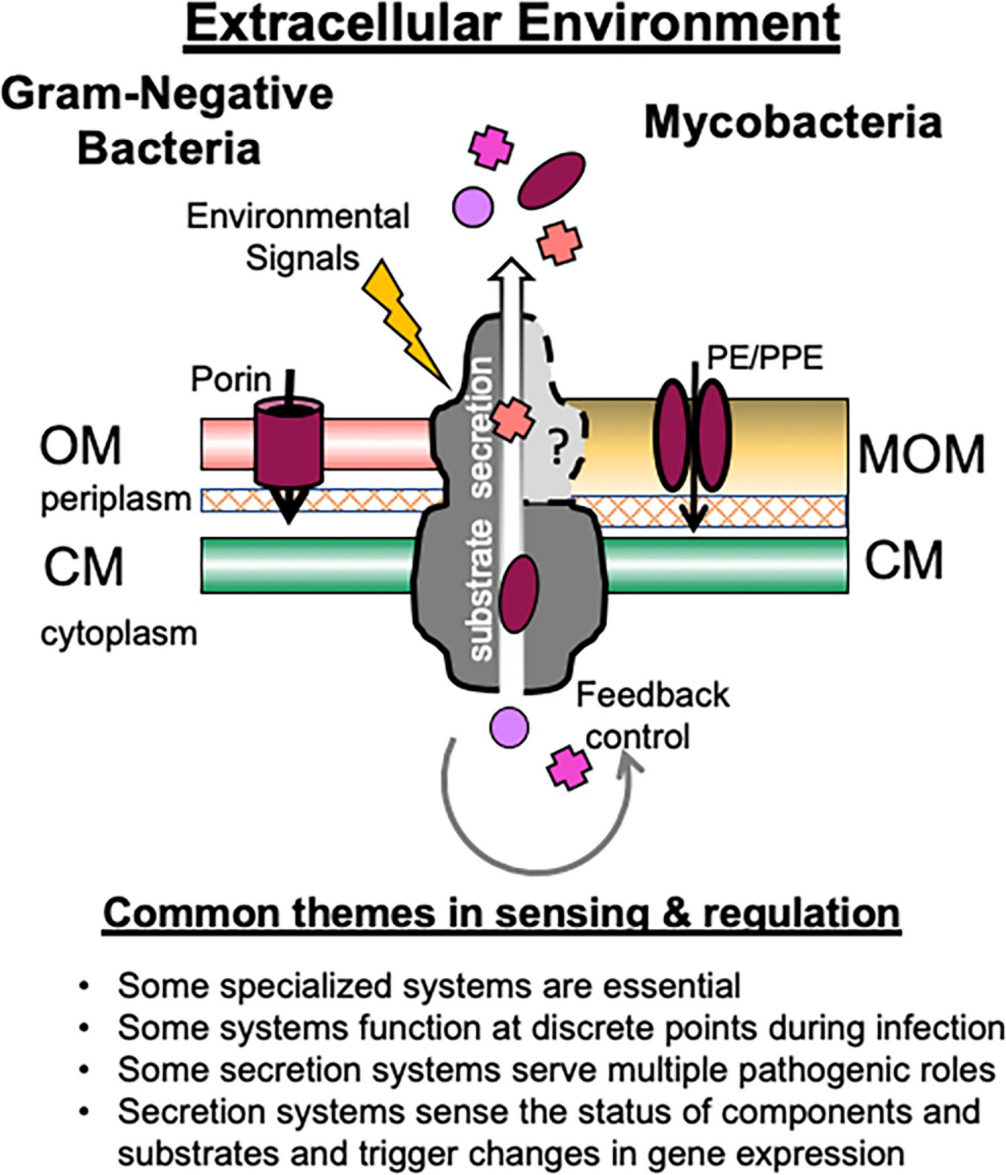
Conserved themes in protein secretion. Gram-negative bacteria and mycobacterial secretion systems also have conserved sensing and regulatory mechanisms (bulleted list). Secretion systems of gram-negative bacteria and mycobacteria both employ feedback regulation (gray arrow) to control the amount of substrates that are produced or secreted (gradient arrow). Some gram-negative bacteria sense and respond to their external environments (lightning bolt). Gram-negative bacteria use porins for solute transport, while mycobacteria use PE/PPE proteins for solute transport. No transporter has yet been identified in the MOM of mycobacteria. CM, cytoplasmic membrane; OM, outer membrane; MOM, mycolic acid outer membrane.

Bacterial secretion systems serve a wide variety of functions. From general cellular processes to specialized pathogenic functions, these secretion systems are critical for pathogenic regulation and adaptation. Despite system conservation and structural similarities, these machines have slight variations in composition and regulation that result in unique signaling mechanisms, adding specificity to function (Fig 1). Each system is one component of a network of secretion systems that are intertwined and interdependent. The complexity of each system adds to bacterial adaptation and pathogenesis. Ultimately, secretion systems provide the bacteria with several inputs of environmental context, ensuring that environmental signals are integrated into the global network for adaptation, enhancing survival and pathogenesis.

## References

[1] Natale P, Bruser T, Driessen AJ. Sec- and Tat-mediated protein secretion across the bacterial cytoplasmic membrane—distinct translocases and mechanisms. Biochim Biophys Acta 2008;1778(9):1735–56. Epub 2007/10/16. doi: 10.1016/j.bbamem.2007.07.015 . 17935691

[2] Green ER, Mecsas J. Bacterial Secretion Systems: An Overview. Microbiol Spectr. 2016;4(1). Epub 2016/03/22. doi: 10.1128/microbiolspec.VMBF-0012-2015 ; PubMed Central PMCID: PMC4804464. 26999395 PMC4804464

[3] Chagnot C, Zorgani MA, Astruc T, Desvaux M. Proteinaceous determinants of surface colonization in bacteria: bacterial adhesion and biofilm formation from a protein secretion perspective. Front Microbiol. 2013;4:303. doi: 10.3389/fmicb.2013.00303 . 24133488 PMC3796261

[4] Lauber F, Deme JC, Lea SM, Berks BC. Type 9 secretion system structures reveal a new protein transport mechanism. Nature. 2018;564(7734):77–82. Epub 2018/11/09. doi: 10.1038/s41586-018-0693-y ; PubMed Central PMCID: PMC6927815. 30405243 PMC6927815

[5] Costa TR, Felisberto-Rodrigues C, Meir A, Prevost MS, Redzej A, Trokter M, et al. Secretion systems in Gram-negative bacteria: structural and mechanistic insights. Nat Rev Microbiol 2015;13(6):343–59. Epub 2015/05/16. doi: 10.1038/nrmicro3456 . 25978706

[6] Palmer T, Finney AJ, Saha CK, Atkinson GC, Sargent F. A holin/peptidoglycan hydrolase-dependent protein secretion system. Mol Microbiol 2021;115(3):345–55. Epub 2020/09/05. doi: 10.1111/mmi.14599 . 32885520

[7] Hagan CL, Silhavy TJ, Kahne D. beta-Barrel membrane protein assembly by the Bam complex. Annu Rev Biochem 2011;80:189–210. Epub 2011/03/05. doi: 10.1146/annurev-biochem-061408-144611 . 21370981

[8] Bansal-Mutalik R, Nikaido H. Mycobacterial outer membrane is a lipid bilayer and the inner membrane is unusually rich in diacyl phosphatidylinositol dimannosides. Proc Natl Acad Sci U S A. 2014;111(13):4958–63. doi: 10.1073/pnas.1403078111 ; PubMed Central PMCID: PMC3977252 24639491 PMC3977252

[9] Brennan PJ, Nikaido H. The envelope of mycobacteria. Annu Rev Biochem. 1995;64:29–63. doi: 10.1146/annurev.bi.64.070195.000333 . 7574484

[10] van Winden VJC, Houben ENG, Braunstein M. Protein Export into and across the Atypical Diderm Cell Envelope of Mycobacteria. Microbiol Spectr. 2019;7(4). Epub 2019/08/11. doi: 10.1128/microbiolspec.GPP3-0043-2018 . 31400094 PMC10957183

[11] Rivera-Calzada A, Famelis N, Llorca O, Geibel S. Type VII secretion systems: structure, functions and transport models. Nat Rev Microbiol 2021;19(9):567–84. Epub 2021/05/28. doi: 10.1038/s41579-021-00560-5 . 34040228

[12] Abdallah AM, Gey van Pittius NC, Champion PA, Cox J, Luirink J, Vandenbroucke-Grauls CM, et al. Type VII secretion—mycobacteria show the way. Nat Rev Microbiol 2007;5(11):883–91. doi: 10.1038/nrmicro1773 . 17922044

[13] Unnikrishnan M, Constantinidou C, Palmer T, Pallen MJ. The Enigmatic Esx Proteins: Looking Beyond Mycobacteria. Trends Microbiol. 2017;25(3):192–204. doi: 10.1016/j.tim.2016.11.004 . 27894646

[14] Bunduc CM, Fahrenkamp D, Wald J, Ummels R, Bitter W, Houben ENG, et al. Structure and dynamics of a mycobacterial type VII secretion system. Nature. 2021;593(7859):445–8. Epub 2021/05/14. doi: 10.1038/s41586-021-03517-z ; PubMed Central PMCID: PMC8131196. 33981042 PMC8131196

[15] Beckham KS, Ciccarelli L, Bunduc CM, Mertens HD, Ummels R, Lugmayr W, et al. Structure of the mycobacterial ESX-5 type VII secretion system membrane complex by single-particle analysis. Nat Microbiol. 2017;2:17047. doi: 10.1038/nmicrobiol.2017.47 . 28394313

[16] Famelis N, Rivera-Calzada A, Degliesposti G, Wingender M, Mietrach N, Skehel JM, et al. Architecture of the mycobacterial type VII secretion system. Nature. 2019;576(7786):321–5. Epub 2019/10/10. doi: 10.1038/s41586-019-1633-1 ; PubMed Central PMCID: PMC6914368. 31597161 PMC6914368

[17] Poweleit N, Czudnochowski N, Nakagawa R, Trinidad DD, Murphy KC, Sassetti CM, et al. The structure of the endogenous ESX-3 secretion system. Elife. 2019;8. Epub 2019/12/31. doi: 10.7554/eLife.52983 ; PubMed Central PMCID: PMC6986878. 31886769 PMC6986878

[18] Gey van Pittius NC, Sampson SL, Lee H, Kim Y, van Helden PD, Warren RM. Evolution and expansion of the *Mycobacterium tuberculosis* PE and PPE multigene families and their association with the duplication of the ESAT-6 (esx) gene cluster regions. BMC Evol Biol. 2006;6:95. doi: 10.1186/1471-2148-6-95 ; PubMed Central PMCID: PMC1660551. 17105670 PMC1660551

[19] Wang Q, Boshoff HIM, Harrison JR, Ray PC, Green SR, Wyatt PG, et al. PE/PPE proteins mediate nutrient transport across the outer membrane of *Mycobacterium tuberculosis*. Science 2020;367(6482):1147–51. Epub 2020/03/07. doi: 10.1126/science.aav5912 . 32139546 PMC11036889

[20] Babu Sait MR, Koliwer-Brandl H, Stewart JA, Swarts BM, Jacobsen M, Ioerger TR, et al. PPE51 mediates uptake of trehalose across the mycomembrane of *Mycobacterium tuberculosis*. Sci Rep. 2022;12(1):2097. Epub 2022/02/10. doi: 10.1038/s41598-022-06109-7 ; PubMed Central PMCID: PMC8826857. 35136132 PMC8826857

[21] Ehtram A, Shariq M, Ali S, Quadir N, Sheikh JA, Ahmad F, et al. Teleological cooption of *Mycobacterium tuberculosis* PE/PPE proteins as porins: Role in molecular immigration and emigration. Int J Med Microbiol 2021;311(3):151495. Epub 2021/03/18. doi: 10.1016/j.ijmm.2021.151495 . 33730677

[22] Pallen MJ. The ESAT-6/WXG100 superfamily—and a new Gram-positive secretion system? Trends Microbiol 2002;10(5):209–12. doi: 10.1016/s0966-842x(02)02345-4 . 11973144

[23] Pajuelo D, Tak U, Zhang L, Danilchanka O, Tischler AD, Niederweis M. Toxin secretion and trafficking by *Mycobacterium tuberculosis*. Nat Commun 2021;12(1):6592. Epub 2021/11/17. doi: 10.1038/s41467-021-26925-1 . 34782620 PMC8593097

[24] Izquierdo Lafuente B, Ummels R, Kuijl C, Bitter W, Speer A. *Mycobacterium tuberculosis* Toxin CpnT Is an ESX-5 Substrate and Requires Three Type VII Secretion Systems for Intracellular Secretion. MBio. 2021;12(2). Epub 2021/03/04. doi: 10.1128/mBio.02983-20 ; PubMed Central PMCID: PMC8092274. 33653883 PMC8092274

[25] Champion MM, Williams EA, Pinapati RS, Champion PA. Correlation of Phenotypic Profiles Using Targeted Proteomics Identifies Mycobacterial Esx-1 Substrates. J Proteome Res. 2014;3(11):5151–64. doi: 10.1021/pr500484w . 25106450 PMC4227905

[26] Wang L, Asare E, Shetty AC, Sanchez-Tumbaco F, Edwards MR, Saranathan R, et al. Multiple genetic paths including massive gene amplification allow *Mycobacterium tuberculosis* to overcome loss of ESX-3 secretion system substrates. Proc Natl Acad Sci U S A. 2022;119(8). Epub 2022/02/24. doi: 10.1073/pnas.2112608119 ; PubMed Central PMCID: PMC8872769. 35193958 PMC8872769

[27] Denise R, Abby SS, Rocha EPC. The Evolution of Protein Secretion Systems by Co-option and Tinkering of Cellular Machineries. Trends Microbiol 2020;28(5):372–86. Epub 2020/04/17. doi: 10.1016/j.tim.2020.01.005 . 32298615

[28] Nagai H, Kubori T. Type IVB Secretion Systems of *Legionella* and Other Gram-Negative Bacteria. Front Microbiol. 2011;2:136. Epub 2011/07/12. doi: 10.3389/fmicb.2011.00136 ; PubMed Central PMCID: PMC3127085. 21743810 PMC3127085

[29] Qiu J, Luo ZQ. *Legionella* and *Coxiella* effectors: strength in diversity and activity. Nat Rev Microbiol 2017;15(10):591–605. Epub 2017/07/18. doi: 10.1038/nrmicro.2017.67 . 28713154

[30] Voth DE, Broederdorf LJ, Graham JG. Bacterial Type IV secretion systems: versatile virulence machines. Future Microbiol. 2012;7(2):241–57. Epub 2012/02/14. doi: 10.2217/fmb.11.150 ; PubMed Central PMCID: PMC3563059. 22324993 PMC3563059

[31] Beare PA, Unsworth N, Andoh M, Voth DE, Omsland A, Gilk SD, et al. Comparative genomics reveal extensive transposon-mediated genomic plasticity and diversity among potential effector proteins within the genus *Coxiella*. Infect Immun. 2009;77(2):642–56. Epub 2008/12/03. doi: 10.1128/IAI.01141-08 ; PubMed Central PMCID: PMC2632050. 19047403 PMC2632050

[32] Voth DE, Heinzen RA. *Coxiella* type IV secretion and cellular microbiology. Curr Opin Microbiol. 2009;12(1):74–80. Epub 2009/01/16. doi: 10.1016/j.mib.2008.11.005 ; PubMed Central PMCID: PMC2670610. 19144560 PMC2670610

[33] Costa TRD, Harb L, Khara P, Zeng L, Hu B, Christie PJ. Type IV secretion systems: Advances in structure, function, and activation. Mol Microbiol. 2021;115(3):436–52. Epub 2020/12/17. doi: 10.1111/mmi.14670 ; PubMed Central PMCID: PMC8026593. 33326642 PMC8026593

[34] Bandyopadhyay P, Lang EA, Rasaputra KS, Steinman HM. Implication of the VirD4 coupling protein of the Lvh type 4 secretion system in virulence phenotypes of *Legionella pneumophila*. J Bacteriol. 2013;195(15):3468–75. Epub 2013/06/05. doi: 10.1128/JB.00430-13 ; PubMed Central PMCID: PMC3719543. 23729650 PMC3719543

[35] Kyrova K, Stepanova H, Rychlik I, Faldyna M, Volf J. SPI-1 encoded genes of *Salmonella typhimurium* influence differential polarization of porcine alveolar macrophages in vitro. BMC Vet Res. 2012;8:115. Epub 2012/07/24. doi: 10.1186/1746-6148-8-115 ; PubMed Central PMCID: PMC3441223. 22817641 PMC3441223

[36] Hapfelmeier S, Stecher B, Barthel M, Kremer M, Muller AJ, Heikenwalder M, et al. The Salmonella pathogenicity island (SPI)-2 and SPI-1 type III secretion systems allow *Salmonella* serovar *typhimurium* to trigger colitis via MyD88-dependent and MyD88-independent mechanisms. J Immunol 2005;174(3):1675–85. Epub 2005/01/22. doi: 10.4049/jimmunol.174.3.1675 . 15661931

[37] Figueira R, Holden DW. Functions of the Salmonella pathogenicity island 2 (SPI-2) type III secretion system effectors. Microbiology. 2012;158(Pt 5):1147–61. doi: 10.1099/mic.0.058115-0 . 22422755

[38] Galan JE. Molecular genetic bases of Salmonella entry into host cells. Mol Microbiol 1996;20(2):263–71. Epub 1996/04/01. doi: 10.1111/j.1365-2958.1996.tb02615.x . 8733226

[39] Simeone R, Bobard A, Lippmann J, Bitter W, Majlessi L, Brosch R, et al. Phagosomal rupture by *Mycobacterium tuberculosis* results in toxicity and host cell death. PLoS Pathog. 2012;8(2):e1002507. doi: 10.1371/journal.ppat.1002507 . 22319448 PMC3271072

[40] Manzanillo PS, Shiloh MU, Portnoy DA, Cox JS. *Mycobacterium Tuberculosis* Activates the DNA-Dependent Cytosolic Surveillance Pathway within Macrophages. Cell Host Microbe. 2012;11(5):469–80. doi: 10.1016/j.chom.2012.03.007 . 22607800 PMC3662372

[41] Mittal E, Skowyra ML, Uwase G, Tinaztepe E, Mehra A, Koster S, et al. *Mycobacterium tuberculosis* Type VII Secretion System Effectors Differentially Impact the ESCRT Endomembrane Damage Response. MBio 2018;9(6):e01765–18. Epub 2018/11/30. doi: 10.1128/mBio.01765-18 . 30482832 PMC6282207

[42] Serafini A, Boldrin F, Palu G, Manganelli R. Characterization of a *Mycobacterium tuberculosis* ESX-3 conditional mutant: essentiality and rescue by Iron and Zinc. J Bacteriol. 2009;191(20):6340–4. doi: 10.1128/JB.00756-09 . 19684129 PMC2753049

[43] Siegrist MS, Unnikrishnan M, McConnell MJ, Borowsky M, Cheng TY, Siddiqi N, et al. Mycobacterial Esx-3 is required for mycobactin-mediated iron acquisition. Proc Natl Acad Sci U S A. 2009;106(44):18792–7. doi: 10.1073/pnas.0900589106 . 19846780 PMC2774023

[44] Ates LS, Ummels R, Commandeur S, van de Weerd R, Sparrius M, Weerdenburg E, et al. Essential Role of the ESX-5 Secretion System in Outer Membrane Permeability of Pathogenic Mycobacteria. PLoS Genet. 2015;11(5):e1005190. Epub 2015/05/06. doi: 10.1371/journal.pgen.1005190 ; PubMed Central PMCID: PMC4418733. 25938982 PMC4418733

[45] Di Luca M, Bottai D, Batoni G, Orgeur M, Aulicino A, Counoupas C, et al. The ESX-5 associated eccB-EccC locus is essential for *Mycobacterium tuberculosis* viability. PLoS ONE. 2012;7(12):e52059. Epub 2013/01/04. doi: 10.1371/journal.pone.0052059 ; PubMed Central PMCID: PMC3524121. 23284869 PMC3524121

[46] Bottai D, Di Luca M, Majlessi L, Frigui W, Simeone R, Sayes F, et al. Disruption of the ESX-5 system of *Mycobacterium tuberculosis* causes loss of PPE protein secretion, reduction of cell wall integrity and strong attenuation. Mol Microbiol. 2012;83(6):1195–209. doi: 10.1111/j.1365-2958.2012.08001.x . 22340629

[47] Bensing BA, Seepersaud R, Yen YT, Sullam PM. Selective transport by SecA2: an expanding family of customized motor proteins. Biochim Biophys Acta. 2014;1843(8):1674–86. Epub 2013/11/05. doi: 10.1016/j.bbamcr.2013.10.019 ; PubMed Central PMCID: PMC4007388. 24184206 PMC4007388

[48] van der Woude AD, Stoop EJ, Stiess M, Wang S, Ummels R, van Stempvoort G, et al. Analysis of SecA2-dependent substrates in *Mycobacterium marinum* identifies protein kinase G (PknG) as a virulence effector. Cell Microbiol 2014;16(2):280–95. Epub 2013/10/15. doi: 10.1111/cmi.12221 . 24119166

[49] Yahr TL, Wolfgang MC. Transcriptional regulation of the *Pseudomonas aeruginosa* type III secretion system. Mol Microbiol 2006;62(3):631–40. Epub 2006/09/26. doi: 10.1111/j.1365-2958.2006.05412.x . 16995895

[50] Barbosa VAA, Lery LMS. Insights into *Klebsiella pneumoniae* type VI secretion system transcriptional regulation. BMC Genomics. 2019;20(1):506. Epub 2019/06/20. doi: 10.1186/s12864-019-5885-9 ; PubMed Central PMCID: PMC6580597. 31215404 PMC6580597

[51] Lazzaro M, Feldman MF, Garcia Vescovi E. A Transcriptional Regulatory Mechanism Finely Tunes the Firing of Type VI Secretion System in Response to Bacterial Enemies. MBio. 2017;8(4). Epub 2017/08/24. doi: 10.1128/mBio.00559-17 ; PubMed Central PMCID: PMC5565961. 28830939 PMC5565961

[52] Ito K, Mori H, Chiba S. Monitoring substrate enables real-time regulation of a protein localization pathway. FEMS Microbiol Lett. 2018;365(11). Epub 2018/05/24. doi: 10.1093/femsle/fny109 . 29790986

[53] Raghavan S, Manzanillo P, Chan K, Dovey C, Cox JS. Secreted transcription factor controls *Mycobacterium tuberculosis* virulence. Nature. 2008;454(7205):717–21. doi: 10.1038/nature07219 . 18685700 PMC2862998

[54] Bosserman RE, Nguyen TT, Sanchez KG, Chirakos AE, Ferrell MJ, Thompson CR, et al. WhiB6 regulation of ESX-1 gene expression is controlled by a negative feedback loop in M*ycobacterium marinum*. Proc Natl Acad Sci U S A. 2017. doi: 10.1073/pnas.1710167114 . 29180415 PMC5740670

[55] Abdallah AM, Weerdenburg EM, Guan Q, Ummels R, Borggreve S, Adroub SA, et al. Integrated transcriptomic and proteomic analysis of pathogenic mycobacteria and their esx-1 mutants reveal secretion-dependent regulation of ESX-1 substrates and WhiB6 as a transcriptional regulator. PLoS ONE 2019;14(1):e0211003. Epub 2019/01/24. doi: 10.1371/journal.pone.0211003 . 30673778 PMC6343904

[56] Solans L, Aguilo N, Samper S, Pawlik A, Frigui W, Martin C, et al. A specific polymorphism in *Mycobacterium tuberculosis* H37Rv causes differential ESAT-6 expression and identifies WhiB6 as a novel ESX-1 component. Infect Immun. 2014;82(8):3446–56. doi: 10.1128/IAI.01824-14 ; PubMed Central PMCID: PMC4136221. 24891105 PMC4136221

[57] Sanchez KG, Ferrell MJ, Chirakos AE, Nicholson KR, Abramovitch RB, Champion MM, et al. EspM Is a Conserved Transcription Factor That Regulates Gene Expression in Response to the ESX-1 System. MBio 2020;11(1). Epub 2020/02/06. doi: 10.1128/mBio.02807-19 . 32019792 PMC7002343

[58] Chirakos AE, Nicholson KR, Huffman A, Champion PA. Conserved ESX-1 Substrates EspE and EspF Are Virulence Factors That Regulate Gene Expression. Infect Immun. 2020;88(12). Epub 2020/09/10. doi: 10.1128/IAI.00289-20 ; PubMed Central PMCID: PMC7671884. 32900815 PMC7671884

[59] Miller VL. Connections between transcriptional regulation and type III secretion? Curr Opin Microbiol. 2002;5(2):211–5. doi: 10.1016/s1369-5274(02)00303-x . 11934620

[60] Diaz MR, King JM, Yahr TL. Intrinsic and Extrinsic Regulation of Type III Secretion Gene Expression in *Pseudomonas Aeruginosa*. Front Microbiol. 2011;2:89. doi: 10.3389/fmicb.2011.00089 ; PubMed Central PMCID: PMC3153048 21833328 PMC3153048

[61] Gonzalo-Asensio J, Malaga W, Pawlik A, Astarie-Dequeker C, Passemar C, Moreau F, et al. Evolutionary history of tuberculosis shaped by conserved mutations in the PhoPR virulence regulator. Proc Natl Acad Sci U S A. 2014;111(31):11491–6. doi: 10.1073/pnas.1406693111 ; PubMed Central PMCID: PMC4128152. 25049399 PMC4128152

[62] Notti RQ, Stebbins CE. The Structure and Function of Type III Secretion Systems. Microbiol Spectr. 2016;4(1). Epub 2016/03/22. doi: 10.1128/microbiolspec.VMBF-0004-2015 ; PubMed Central PMCID: PMC4804468. 26999392 PMC4804468

[63] Bachmann V, Kostiuk B, Unterweger D, Diaz-Satizabal L, Ogg S, Pukatzki S. Bile Salts Modulate the Mucin-Activated Type VI Secretion System of Pandemic *Vibrio cholerae*. PLoS Negl Trop Dis. 2015;9(8):e0004031. Epub 2015/09/01. doi: 10.1371/journal.pntd.0004031 ; PubMed Central PMCID: PMC4552747. 26317760 PMC4552747

[64] Conrad WH, Osman MM, Shanahan JK, Chu F, Takaki KK, Cameron J, et al. Mycobacterial ESX-1 secretion system mediates host cell lysis through bacterium contact-dependent gross membrane disruptions. Proc Natl Acad Sci U S A. 2017;114(6):1371–6. doi: 10.1073/pnas.1620133114 . 28119503 PMC5307465

